# Suitability of the muscle O_2_ resaturation parameters most used for assessing reactive hyperemia: a near-infrared spectroscopy study

**DOI:** 10.1590/1677-5449.200143

**Published:** 2021-05-24

**Authors:** Gustavo Vieira de Oliveira, Mônica Volino-Souza, Renata Leitão, Vivian Pinheiro, Carlos Adam Conte-Júnior, Thiago Silveira Alvares

**Affiliations:** 1 Universidade Federal do Rio de Janeiro – UFRJ, Macaé, RJ, Brasil.

**Keywords:** cardiovascular physiology, capillary endothelial cells, vascular disease, hypertension, aging, fisiologia cardiovascular, células endoteliais capilares, doenças vasculares, hipertensão arterial, envelhecimento

## Abstract

**Background:**

There is a spectrum of possibilities for analyzing muscle O_2_ resaturation parameters for measurement of reactive hyperemia in microvasculature. However, there is no consensus with respect to the responsiveness of these O_2_ resaturation parameters for assessing reactive hyperemia.

**Objectives:**

This study investigates the responsiveness of the most utilized muscle O_2_ resaturation parameters to assess reactive hyperemia in the microvasculature of a clinical group known to exhibit impairments of tissue O_2_ saturation (StO_2_).

**Methods:**

Twenty-three healthy young adults, twenty-nine healthy older adults, and thirty-five older adults at risk of cardiovascular disease (CVD) were recruited. Near-infrared spectroscopy (NIRS) was used to assess StO_2_ after a 5-min arterial occlusion challenge and the following parameters were analyzed: StO_2slope_10s_, StO_2slope_30s_, and StO_2slope_until_baseline_ (upslope of StO_2_ over 10s and 30s and until StO_2_ reaches the baseline value); time to StO_2baseline_ and time to StO_2max_ (time taken for StO_2_ to reach baseline and peak values, respectively); ∆StO_2reperfusion_ (the difference between minimum and maximum StO_2_ values); total area under the curve (StO_2AUCt_); and AUC above the baseline value (StO_2AUC_above_base_).

**Results:**

Only StO_2slope_10s_ was significantly slower in older adults at risk for CVD compared to healthy young individuals (p < 0.001) and to healthy older adults (p < 0.001). Conversely, time to StO_2max_ was significantly longer in healthy young individuals than in older adult at CVD risk.

**Conclusions:**

Our findings suggest that StO_2slope_10s_ may be a measure of reactive hyperemia, which provides clinical insight into microvascular function assessment.

## Resumo

**Contexto:**

Existe um espectro de possibilidades na análise dos parâmetros de ressaturação de O_2_ muscular como uma medida de hiperemia reativa na microvasculatura. No entanto, não há consenso com relação à responsividade desses parâmetros de ressaturação de O_2_ para avaliação de hiperemia reativa.

**Objetivos:**

Este estudo investigou a capacidade de resposta dos parâmetros de ressaturação muscular de O_2_ mais utilizados para avaliar a hiperemia reativa na microvasculatura de um grupo clínico conhecido por apresentar comprometimento da saturação de O_2_ (StO_2_).

**Métodos:**

Foram recrutados 23 jovens saudáveis, 29 idosos saudáveis e 35 idosos com risco para doença cardiovascular. A espectroscopia no infravermelho próximo foi usada para avaliar a StO_2_ após um teste de oclusão arterial de 5 minutos, no qual os seguintes parâmetros foram analisados: StO_2slope_10s_, StO_2slope_30s_ e StO2_slope_until_baseline_ (inclinação da StO_2_ em 10 s, 30 s e até StO_2_ atingir valores basais); tempo para StO_2baseline_ e tempo para StO_2máx_ (o tempo necessário para StO_2_ atingir os valores da linha de base e o máximo, respectivamente); ∆StO_2reperfusão_ (a diferença entre o valor de StO_2mínimo_ e StO_2máximo_); área total sob a curva (StO_2AUCt_); e área sob a curva acima do valor da linha de base (StO_2AUC_above_base_).

**Resultados:**

Apenas StO_2slope_10s_ foi significativamente mais lento em idosos em risco de doença cardiovascular comparados com indivíduos jovens saudáveis (p < 0,001) e idosos saudáveis (p < 0,001). Por outro lado, o tempo para StO_2max_ foi significativamente maior em indivíduos jovens saudáveis do que em idosos em risco de doença cardiovascular.

**Conclusões:**

Nossos achados sugerem que StO_2slope_10s_ pode ser uma medida de hiperemia reativa, que fornece informações clínicas sobre a avaliação da função microvascular.

## INTRODUCTION

Endothelial dysfunction is an abnormality of the vascular system that predicts a cardiovascular event.^1^^,^ 2 Assessment of reactive hyperemia in microcirculation has been used as a measure of microvascular function.^3^ Microvascular dysfunction may lead to rarefaction of downstream vessels and consequent reduction in the number of capillaries, contributing to hypertension and cardiovascular events.^4^ Moreover, downstream arterial disease may reduce muscle blood supply, leading to fatigue, cramp, discomfort, or pain in limbs during daily activities.^5^ Therefore, measuring microvascular function may be important for identification of pathophysiologic mechanisms that impair adequate tissue blood perfusion.

Over the past decades, near-infrared spectroscopy (NIRS) combined with a vascular occlusion test (VOT) has been used for assessment of tissue O_2_ saturation (StO_2_). Since NIRS detects changes in oxygenated and deoxygenated hemoglobin in the tissues using absorption of near-infrared light at a specific wavelength,^6^^,^ 7 oxygenation kinetics in the microcirculation (i.e., arterioles, venules, and capillaries) can be noninvasively assessed and used to measure reactive hyperemia in microcirculation.^6^^,^ 8

Performing a VOT with an NIRS device fitted to the limb of interest, the StO_2_ signal follows a downward path during the cuff inflation phase and then StO_2_ goes rapidly upward immediately after cuff deflation (reperfusion phase).^8^^-^ 10 The sudden increase in the StO_2_ signal during reperfusion enables various StO_2_ parameters to be calculated, which, in general, are interpreted as measures of microvascular function.^3^ The majority of StO_2_ parameters adopted in previous studies include reperfusion rate (i.e., upslope of the StO_2_ signal during the initial 10 s and 30 s, and until the StO_2_ signal reaches baseline values),^11^^-^ 13 magnitude of reperfusion (i.e., total area under the curve [AUC] of StO_2_ and the difference between the lowest and highest StO_2_ value),^4^^,^ 13 ^-^ 17 and others (e.g., time to StO_2_ baseline, time to StO_2_ maximum, and AUC above the baseline).^13^^,^ 15 ^,^ 18 ^,^ 19

Despite a range of possibilities for interpretation of muscle O_2_ resaturation parameters, the lack of standardization of which NIRS parameters are used during the reperfusion phase makes comparisons between studies difficult and/or may even lead to misinterpretation of results. For example, since the time to maximum StO_2_ parameter is calculated as the time elapsed from release of the cuff to the maximum StO_2_ value during reperfusion, individuals with higher O_2_ extraction capacity (i.e., healthy young individuals) might exhibit a longer time to maximum StO_2_. In other words, in individuals with higher O_2_ extraction, the distance between the lowest StO_2_ value reached during cuff inflation and the highest StO_2_ reached during the cuff deflation is longer, thereby influencing calculation of some NIRS parameters, such as time to StO_2_ maximum and even area under the reperfusion curve. Therefore, the present study investigates the usefulness of the muscle O_2_ resaturation parameters most utilized to assess reactive hyperemia in a clinical group known to exhibit impairments in muscle StO_2_. It was hypothesized that some NIRS parameters might be more sensitive than others (mainly those influenced by muscle O_2_ extraction) for detecting abnormal reactive hyperemia in a clinical sample.

## METHODS

### Participants

Twenty**-**three healthy young individuals, 29 healthy older adults, and 35 older adults at risk of CVD were recruited to participate in the study. For the younger group, inclusion criteria were being healthy (not presenting CVD risk factors) and age < 35 years. For the healthy older adult group, individuals had to be healthy and ≥ 60 years of age. The inclusion criteria for older adults at high risk for CVD were age ≥ 60 years and possessing at least four CVD risk factors, according to a previous study demonstrating that presence of three or more CVD risk factors is related to microvascular dysfunction.^10^^,^ 20 The following CVD risk factors were used as inclusion criteria: triglycerides ≥ 150 mg/dL; total-cholesterol ≥ 200 mg/dL; LDL-cholesterol ≥ 150 mg/dL, HDL-cholesterol ≤ 40 mg/dL for men and ≤ 50 mg/dL for women; high-blood glucose ≥ 120 mg/dL and/or taking oral hypoglycemic medications; and high systolic blood pressure (SBP) ≥ 135 mm Hg, and/or high diastolic blood pressure (DBP) ≥ 90 mm Hg, and/or taking anti-hypertensive medications. The exclusion criteria included human immunodeficiency virus (HIV); cancer; rheumatoid arthritis; smoking; and chronic obstructive pulmonary disease; as well as participants not being engaged in any exercise program. All experimental procedures were performed after explaining the nature of the study and obtaining written consent from participants. The study was conducted in accordance with declaration of Helsinki ethical standards and approved by the institutional ethics committee at the Universidade Federal do Rio de Janeiro (UFRJ), Macaé, RJ, Brasil. (N^o^. 1.489.668).

### Experimental protocol

After 12 h fasting, the participants arrived at the laboratory, where they underwent anthropometric measurement (weight, height, and forearm skin-fold) before blood samples were taken. Blood samples were collected only at baseline in order to determine the CVD risk factors (i.e., fasting blood glucose, triglycerides, cholesterol, etc.) of the participants enrolled on the study. Afterwards, participants rested for 10 min in the supine position on an examination table, followed by assessment of the NIRS parameters for level of muscle oxygen saturation. The experimental procedures were conducted between 8:00 and 10:00 a.m. and participants were recommended to avoid caffeine and foods rich in nitrates and nitrites for 24 h before the laboratory visit.

### Tissue O_2_ saturation measurement

**Figure 1:**
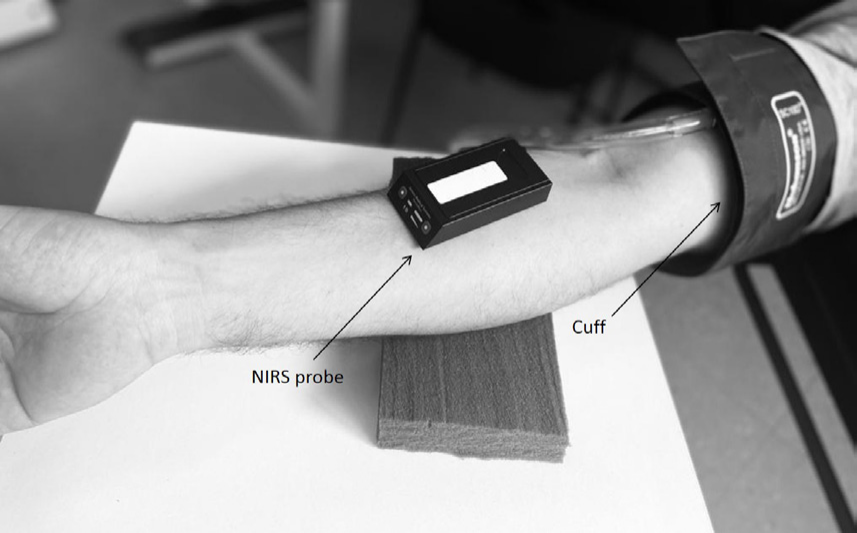
Cuff and near-infrared spectroscopy (NIRS) probe on skin overlying forearm flexor muscles. Note that in the picture the NIRS probe is shown without the black vinyl sheet and the elastic tensor band that cover it, for the purposes of illustrating the probe location in the image.

**Figure 2:**
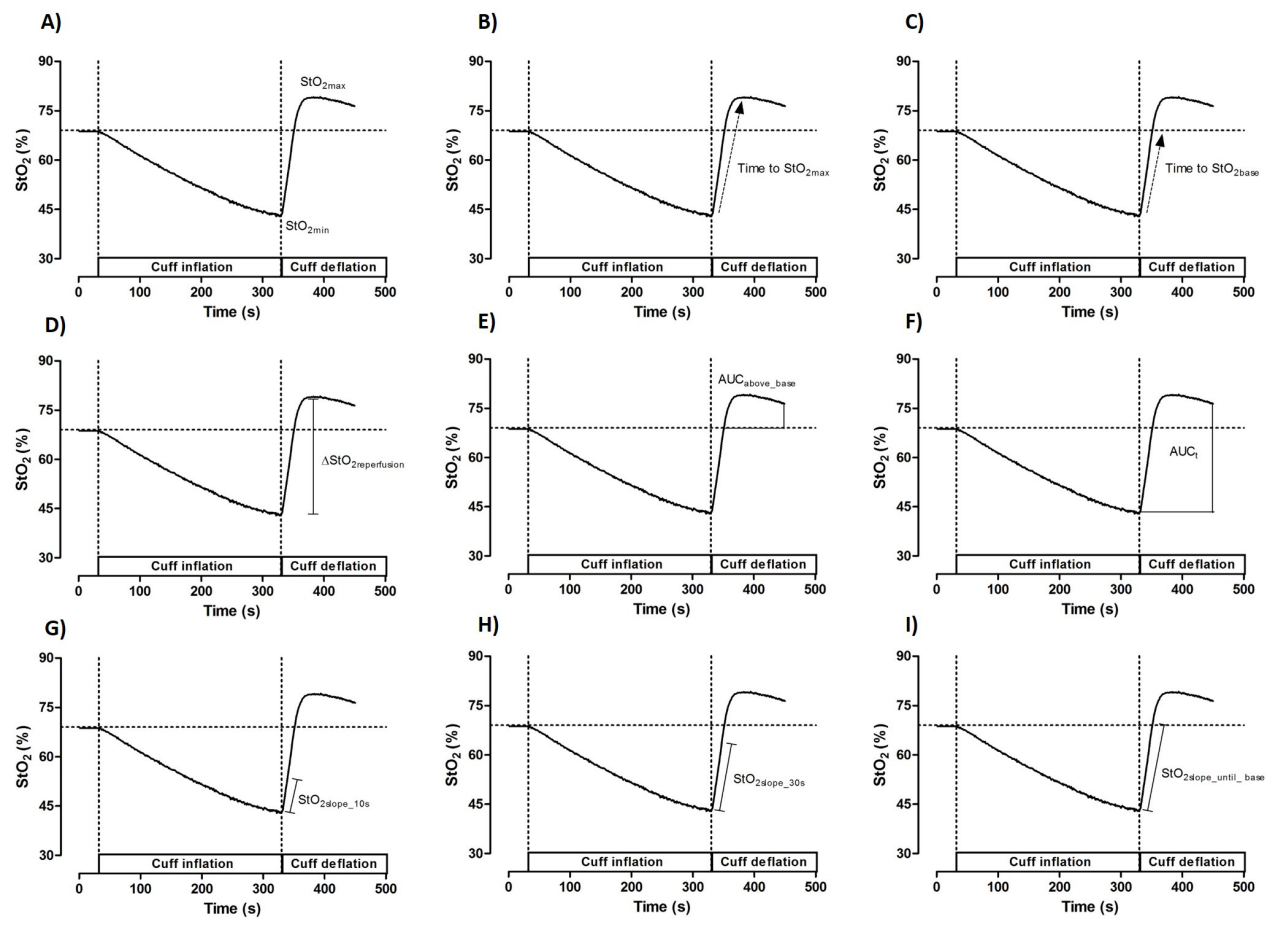
Near-infrared spectroscopy (NIRS)-derived tissue O_2_ saturation (StO_2_) signal analysis during cuff deflation phase (reperfusion). (A) The lowest StO_2_ value reached during cuff inflation (StO_2min_) and the highest StO_2_ value reached following cuff deflation (StO_2max_); (B) time for the StO_2_ signal to reach the peak after cuff release (time to StO_2max_); (C) time for the StO_2_ signal to reach the pre-occlusion baseline values after cuff release (time to StO_2base_); (D) difference between the StO_2min_ and StO_2max_ after cuff deflation (ΔStO_2reperfusion_); (E) area under the reperfusion curve (AUC) above the baseline (StO_2AUCabove_base_); (F) the total AUC over 2 minutes of reperfusion (StO_2AUCt_); (G) upslope of StO_2_ signal over a 10 s reperfusion window immediately following cuff deflation (StO_2slope_10s_); (H) upslope of StO_2_ signal over a 30 s reperfusion window immediately following cuff deflation (StO_2slope_30s_); and (I) upslope of StO_2_ signal over the time elapsed between StO_2min_ and pre-occlusion baseline StO_2_ values.

Tissue O_2_ saturation (%StO_2_) was assessed using an NIRS system (PortaMon, Artinis, Medical Systems) connected to a personal computer via Bluetooth for data acquisition (10 Hz). Analogue‐to‐digital conversion and subsequent analysis of the raw data (i.e., no filter was used) was conducted using native software (Oxysoft version 2.1.6; Artinis Medical Systems), as previously described by Oliveira et al.^7^^,^ 10 In brief, the NIRS device was placed on the forearm flexor muscle (flexor carpi radialis), 2 cm below the medial epicondyle of the humerus, and muscle %StO_2_ was recorded continuously throughout the VOT (30 s at baseline, 5 min of occlusion, and 2 min of reperfusion). The cuff was placed over the arm, 2 cm above the medial epicondyle of the humerus and a cuff pressure of 250 mm Hg was used during the occlusion period (Figure 1). To evaluate muscle StO_2_ during ischemia and reperfusion, the following NIRS parameters were used in the statistical analysis: *(i)* StO_2_ during baseline (StO_2base_, %) was calculated as the average over the 30 s before cuff occlusion; *(ii)* the lowest StO_2_ value reached during cuff inflation (StO_2min_) and the highest StO_2_ value reached following cuff deflation (StO_2max_); *(iii)* time for the StO_2_ signal to reach its peak after cuff release (time to StO_2max_); *(iv)* time for the StO_2_ signal to reach the pre-occlusion baseline value after cuff release (time to StO_2base_); *(v)* difference between StO_2min_ and the StO_2max_ after cuff deflation (ΔStO_2reperfusion_); *(vi)* the area under the reperfusion curve (AUC) above the baseline (StO_2AUCabove_base_); *(vii)* the total AUC over 2 minutes of reperfusion (StO_2AUCt_); *(viii)* upslope of StO_2_ signal over a 10 s reperfusion window immediately following cuff deflation (StO_2slope_10s_); *(ix)* upslope of StO_2_ signal over a 30 s reperfusion window immediately following cuff deflation (StO_2slope_30s_); and *(x)* upslope of StO_2_ signal over the time elapsed between StO_2min_ and reaching pre-occlusion baseline StO_2_ values (Figure 2).

### Statistical analysis

One-way ANOVA was used to identify significant differences between the characteristics of the participants at baseline and tissue StO_2_ parameters during the VOT. An a priori power analysis was conducted (G*Power v. 3.0.1) for a specific test (ANOVA: Fixed effects, omnibus, one-way). Based on a statistical power (1 – β) of 0.80, an effect size of 0.46, and an overall significance level of 0.05, 51 participants would be needed to detect a statistical difference. The effect size was based on a previous study that found d=0.93 (or f=0.46; d=2f) when assessing reperfusion rates in older individuals and young participants.^17^ The present study enrolled 87 participants. Additional post hoc tests with Bonferroni adjustment were performed when appropriate. Statistical significance was set at a P value ≤ 0.05 and the results were expressed as mean ± standard deviation (SD). A commercially available statistical package (IBM SPSS Statistics version 22 for Mac, Armonk, N.Y., USA) was used for statistical analysis. The figure was designed using GraphPad Prism 5.0.

## RESULTS

Baseline characteristics of the participants and medications used are shown in Table 1. Healthy young individuals exhibited significant lower body mass index than healthy older adults (P = 0.029) and than older adults at risk for CVD (P = 0.016). Blood glucose (P < 0.001), total cholesterol (P = 0.004), triglycerides (P = 0.049), and systolic blood pressure (P = 0.001) were significantly higher in older adults at risk for CVD than in healthy young individuals. Moreover, blood glucose (P < 0.001), triglycerides (P = 0.026), and systolic blood pressure (P = 0.012) were significantly higher than in healthy older adults.

**Table 1:** Baseline characteristics of the participants.

**Demographics**	**Young healthy**	**Older healthy**	**Older, at risk of CVD**
N	23	29	35
Male/female	14/9	13/16	14/21
Age (years)	28.4 ± 5.9	67.6 ± 5.8	68.4 ± 8.2
Weight (kg)	74.8 ± 9.3	77.9 ± 13.8	72.6 ± 11.2
Height (cm)	171.8 ± 0.1	168.4 ± 0.8	169.5 ± 0.6
Body mass index (kg/m^2^)	25.2 ± 1.8	30.1 ± 4.1*	30.1 ± 8.8*
Forearm skin fold thickness (mm)	2.1 ± 0.4	2.3 ± 0.6	2.3 ± 0.8
**Biochemistry**			
Blood glucose (mg/dL)	87.91 ± 6.9	90.2 ± 13.4	123.9 ± 41.8*†
Total cholesterol (mg/dL)	157.3 ± 30.1	170.8 ± 30.1	190.3 ± 44.3*
LDL-cholesterol (mg/dL)	121.9 ± 33.1	110.2 ± 31.6	125.0 ± 42.2
HDL-cholesterol (mg/dL)	39.7 ± 8.9	44.7 ± 10.9	37.9 ± 13.4
Triglycerides (mg/dL)	121.2 ± 33.7	112.3 ± 29.4	146.5 ± 64.9*†
**Clinical**			
SBP (mm Hg)	122.1 ± 10.1	125.8 ± 4.4	136.8 ± 20.6*†
DBP (mm Hg)	80.3 ± 5.2	80.8 ± 9.1	81.6 ± 9.2
**Medications used**	**n**	**n**	**n**
ACE-i/ARB	-	-	22
Diuretic	-	-	17
*α*-blocker	-	-	1
*β*-blocker	-	-	10
CCB	-	-	6
Antiplatelet drugs	-	-	5
Statins	-	-	12

N = number; CVD = cardiovascular disease; SBP = systolic blood pressure; DBP = diastolic blood pressure; ACE-I = angiotensin converting enzyme inhibitors; ARB = angiotensin receptor blockers; CCB = calcium channel blockers.

* Significantly different from healthy young group;

† Significantly different from healthy older adults. Statistical significance was set at the 0.05 level of confidence. Values were expressed as means ± standard deviation.

Muscle StO_2_ saturation parameters are shown in Table 2. Significantly slower StO_2slope___10s_ (P < 0.001), and StO_2slope___30s_ (P < 0.001), and blunted ΔStO_2reperfusion_ (P < 0.001), StO_2AUCabove_base_ (P < 0.001), StO_2AUCt_ (P < 0.001), StO_2max_ (P = 0.035), and StO_2 min_ (P < 0.001) were observed in older adults at CVD risk compared to healthy young individuals. Additionally, StO_2slope___10s_ was significantly slower (P < 0.001) in older adults at risk for CVD compared to their healthy counterparts. Time to StO_2max_ was significantly slower (P = 0.047) in healthy young individuals than older adults at risk of CVD.

**Table 2:** Muscle O_2_ saturation parameters in healthy young individuals and older adults with cardiovascular disease (CVD) risk factors.

Variables	Young healthy	Older healthy	Older, at risk of CVD
StO_2base_ (%)	69.39 ± 5.10	70.91 ± 3.45	70.28 ± 3.52
StO_2slope___10s_ (%.s^-1^)	1.36 ± 0.48	1.02 ± 0.34*	0.48 ± 0.44*†
StO_2slope___30s_ (%.s^-1^)	1.21 ± 0.35	0.90 ± 0.31*	0.75 ± 0.27*
StO_2slope_until_base_ (%.s^-1^)	1.24 ± 0.41	1.09 ± 0.37	0.98 ± 0.40
Time to StO_2base_ (s)	22.39 ± 11.53	20.34 ± 6.21	19.00 ± 6.55
Time to StO_2max_ (s)	54.17 ± 19.58	46.82 ± 16.64	44.68 ± 14.76*
ΔStO_2reperfusion_ (%)	36.04 ± 9.91	26.30 ± 8.24*	22.79 ± 7.46*
StO_2_AUC_above_base_ (%. s)	1137.77 ± 321.99	860.31 ± 310.43*	699.62 ± 309.78*
StO_2_AUCt (%. s)	3578.34 ± 1046.77	2663.58 ± 765.86*	2348.91 ± 739.57*
StO_2max_ (%)	79.98 ± 5.11	78.73 ± 3.49	77.24 ± 3.41*
StO_2min_ (%)	44.00 ± 10.39	52.33 ± 7.40*	54.44 ± 8.11*

* Significantly different from young group;

† Significantly different from healthy older adults. Statistical significance was set at the 0.05 level of confidence. Values were expressed as means ± SD. Base = baseline; AUC = area under the curve; StO_2_ = tissue O_2_ saturation

Significant slower StO_2slope_30s_ (P = 0.003) and blunted ΔStO_2reperfusion_ (P < 0.001), StO_2AUCabove_base_ (P = 0.006), StO_2AUCt_ (P = 0.001), and StO_2 min_ (P = 0.002) were found in healthy older adults compared to healthy young people. No significant differences in StO_2base_, time to StO_2base_, or StO_2slope_until_base_ were found between groups (P > 0.05).

## DISCUSSION

Given the widespread utilization of NIRS devices and the spectrum of possibilities for analyzing muscle O_2_ resaturation parameters as a measurement of reactive hyperemia in the microvasculature of different populations, the present study investigated the effectiveness of the O_2_ resaturation parameters most frequently used for evaluating reactive hyperemia in healthy older adults or older adults with CVD risk factors. It was hypothesized that analyzing the effectiveness of the most widespread NIRS parameters for detecting aging and CVD risk factors might help with development of criteria for establishing an appropriate set of muscle StO_2_ parameters in order to facilitate the choice between different methodologies of NIRS analysis.

Muscle O_2_ resaturation rate parameters (StO_2slope___10s_, StO_2slope___30s_, and StO_2slope___until_base_) are largely adopted to assess reactive hyperemia in hypertensive individuals,^4^ gestational diabetic women,^14^ and older adults at risk for CVD disease,^7^^,^ 10 ^,^ 17 among other clinical groups.^13^^,^ 18 Our findings demonstrated that only StO_2slope___10s_ was significantly slower in older adults with CVD risk factors compared to healthy older adults and to healthy young individuals, suggesting that StO_2slope_10s_ may be a more sensitive NIRS parameter for assessing reactive hyperemia. The StO_2slope___10s_ has typically been adopted to assess O_2_ resaturation rate, since previous studies have reported that this parameter is correlated with reactive hyperemia measured in the brachial artery^20^ and with FMD response.^8^^,^ 11 It has been postulated that the StO_2slope___10s_ may be a stimulus for FMD response given that FMD is evoked by increasing blood flow after cuff release.^8^^,^ 20 ^,^ 21 In addition, utilizing the StO_2slope_10s_ may be considered an appropriate way to analyze O_2_ reperfusion rate given the linearity of the StO_2_ signal over the initial 10 s after cuff release.^22^

Time to StO_2max_ may be mainly affected by the degree of muscle O_2_ extraction (StO_2min_), since this recovery time parameter measures the time interval between the StO_2min_ (the lowest StO_2_ value reached during cuff inflation) and StO_2max_ (the highest StO_2_ value reached during cuff deflation). Therefore, individuals with higher muscle O_2_ extraction capacity (mitochondrial function) will reach a lower StO_2_ value during the cuff inflation phase (StO_2min_), which makes the time to StO_2max_ longer for those individuals with higher O_2_ extraction capacity.^17^^,^ 23 In support of this observation, a significantly longer time to StO_2max_ was found in healthy individuals compared to older adults at CVD risk, which may have been the result of a lower StO_2_ value reached during cuff inflation (StO_2min_), combined with the higher StO_2_ value reached during the cuff deflation phase (StO_2max_).

Previous studies have failed to show significant differences in time to StO_2max_ in individuals with metabolic syndrome^24^ and older adults at risk for CVD.^10^ However, Kragelj et al.^18^ found a significantly longer time to StO_2max_ in individuals with peripheral vascular disease (a clinical population) compared to healthy participants. Interestingly, the groups investigated in their study^18^ did not exhibit a significant difference in O_2_ extraction parameters, which may explain the expected longer time to StO_2max_ in individuals with peripheral vascular disease. Therefore, adoption of the recovery time parameters (i.e., time to StO_2max_) should be recommended when the clinical population evaluated is not assumed to exhibit impairment in muscle O_2_ extraction; otherwise, data interpretation and comparison may be rendered incorrect.

The current study also demonstrated a significant reduction in ΔStO_2reperfusion_ and StO_2AUCt_ in healthy older adults and those with CVD risk factors, compared to healthy young individuals. However, no significant difference was observed in these parameters in healthy older adults compared to older adults at risk for CVD, suggesting that ΔStO_2reperfusion_ and StO_2AUCt_ are not sensitive parameters for detecting the effect of CVD risk factors on reactive hyperemia. The ΔStO_2reperfusion_ and StO_2AUCt_ parameters can be interpreted as measures of the magnitude of O_2_ resaturation, since the lowest and highest StO_2_ value (amplitude) within the reperfusion phase (cuff deflation) are considered for calculating these parameters. However, it is important to note that, in common with time to StO_2max_, these parameters may also be affected by parameters of muscle O_2_ extraction during cuff deflation (i.e. StO_2min_).

Corroborating this idea, Rosenberry et al.^17^ demonstrated that the impaired StO_2AUCt_ observed in older adults at CVD risk in comparison to healthy young individuals was eliminated when the level of muscle O_2_ extraction (StO_2min_) was standardized by varying occlusion times (3-, 4-, and 5-min cuff occlusion) across the two age groups. It is likely that differences between ΔStO_2reperfusion_ and StO_2AUCt_ parameters do not only necessarily reflect impairment in O_2_ resaturation magnitude (reactive hyperemia), but also in muscle O_2_ extraction during occlusion (mitochondrial dysfunction).^7^^,^ 17 In light of these findings, since NIRS continuously monitors muscle O_2_ saturation throughout the VOT, assessment of ΔStO_2reperfusion_ and StO_2AUCt_ could be performed controlling the level of tissue ischemia (varying the occlusion times),^17^ which would be crucial to better interpret ΔStO_2reperfusion_ and StO_2AUCt_. Alternatively, AUC_above_base_ is not affected by muscle StO_2min_ and its utilization might be recommendable.

### Experimental considerations

Of note, the NIRS system is a relevant tool that can be easily used during vascular surgery, as it continuously assesses muscle O_2_ saturation. For example, a previous study conducted at our laboratory showed that prolonged occlusion of limb blood flow (i.e., tourniquet application during limb surgery) causes transient microvascular dysfunction that can be assessed by NIRS‐derived reperfusion upslope.^25^ Additionally, this study also demonstrated that using a local nitric oxide donor (transdermal nitroglycerin) administered during prolonged forearm cuff occlusion protected against this microvascular dysfunction,^25^ which is relevant for the field of vascular surgery. Moreover, NIRS systems have been widely utilized to detect impaired microvascular reactivity in many clinical populations, such as hypertension, older adults at risk for CVD, and patients with metabolic syndrome.^4^^,^ 10 ^,^ 24

Although NIRS systems are largely utilized to assess oxygenation of the forearm muscles, one limitation of this technique is the discrete volume of a specific muscle (2 to 6 cm) and the superficial area of skeletal muscle that NIRS examines.^26^ Furthermore, another important factor that should be considered is that adipose tissue thickness can affect NIR light penetration into the muscle of interest and, consequently, the muscle oxygenation levels measured. However, it is important to point out that no significant differences were observed in skin fold thickness between groups, ensuring that the depth of light penetration (~18 mm) sufficiently interrogates the forearm flexor muscles.^26^^,^ 27 Additionally, it is noteworthy that all participants in this study did not undergo a clinical examination by a clinician prior to muscle oxygenation measurements. Although we are aware that it would be important for all participants to undergo clinical examination, the main purpose of this study was to investigate the effectiveness of tissue oxygenation parameters in detecting impaired reactive hyperemia in the microvasculature of a population previously known to have microvascular damage.^3^^,^ 4 ^,^ 7 ^,^ 9 Therefore, the absence of clinical examination does not compromise the results of this study.

In conclusion, the findings of this study suggest that StO_2slope_10s_ was the most sensitive NIRS parameter for assessing reactive hyperemia. Additionally, the magnitude of reperfusion (i.e., StO_2AUCt_) and recovery time (i.e., time to StO_2max_) parameters should be interpreted with caution when assessing clinical populations, since muscle O_2_ extraction may affect those parameters. The results of the present study will be helpful for future studies that investigate the effect of an external factor (disease, means of treatment, etc.) on muscle O_2_ resaturation parameters.
